# What is metabolic syndrome, and why are children getting it?

**DOI:** 10.1111/nyas.12030

**Published:** 2013-01-28

**Authors:** Ram Weiss, Andrew A Bremer, Robert H Lustig

**Affiliations:** 1 Department of Pediatrics, Hadassah Hebrew University School of MedicineJerusalem, Israel; 2 Department of Pediatrics, Vanderbilt University School of MedicineNashville, Tennessee; 3 Department of Pediatrics, University of CaliforniaSan Francisco; 4 The Philip R. Lee Institute for Health Policy Studies, University of CaliforniaSan Francisco

**Keywords:** metabolic syndrome, obesity, diet, insulin resistance, reactive oxygen species

## Abstract

Metabolic syndrome comprises a cluster of cardiovascular risk factors (hypertension, altered glucose metabolism, dyslipidemia, and abdominal obesity) that occur in obese children. However, metabolic syndrome can also occur in lean individuals, suggesting that obesity is a marker for the syndrome, not a cause. Metabolic syndrome is difficult to define, due to its nonuniform classification and reliance on hard cutoffs in the evaluation of disorders with non-Gaussian distributions. Defining the syndrome is even more difficult in children, owing to racial and pubertal differences and lack of cardiovascular events. Lipid partitioning among specific fat depots is associated with insulin resistance, which can lead to mitochondrial overload and dysfunctional subcellular energy use and drive the various elements of metabolic syndrome. Multiple environmental factors, in particular a typical Western diet, drive mitochondrial overload, while other changes in Western society, such as stress and sleep deprivation, increase insulin resistance and the propensity for food intake. These culminate in an adverse biochemical phenotype, including development of altered glucose metabolism and early atherogenesis during childhood and early adulthood.

## Introduction

The rise in the prevalence of obesity in children and adolescents is one of the most alarming public health issues facing the world today.^1^ While this rise seems to have leveled in some parts of the world,^2^ in many others (especially developing countries) it has not, and the prevalence of pediatric metabolic syndrome appears to be increasing.

Childhood obesity can be a harbinger of future health disorders. Childhood obesity tends to track into adulthood: 85% of obese children become obese adults.^3^,^4^ Obese toddlers have an odds ratio of 1.3 for becoming obese adults, while obese teenagers have an odds ratio of 17.5.^5^ Childhood obesity is associated with significant health problems and is an early risk factor for much of adult morbidity and mortality.^6^,^7^ While children rarely develop true cardiovascular events, early evidence of accelerated atherogenesis can be detected. Those who remain obese as adults have a significant risk for the development of type 2 diabetes (T2DM), hypertension, dyslipidemia, and atherosclerotic cardiovascular disease (CVD). This cluster of diseases and disorders is collectively termed *metabolic syndrome*.

Metabolic syndrome affects over 25% of the adult population of the United States.^8^ Controversy exists regarding the various definitions of the syndrome and the ability of the syndrome to predict future adverse cardiometabolic events in a manner surpassing other well-described risk factors. Despite this, there can be little controversy regarding the current national and worldwide epidemic of obesity, and the links between risk factors in youth and subsequent adult cardiometabolic disease.^9^,^10^ Metabolic syndrome is associated with many clinical conditions besides CVD and T2DM, including chronic low-grade inflammation, oxidative stress, hyperuricemia, hypertension, dyslipidemia, hyperandrogenism and polycystic ovary syndrome, hepatic steatosis and nonalcoholic fatty liver disease (NAFLD), impaired glucose tolerance, obstructive sleep apnea (OSA), hypogonadism, vascular dementia and Alzheimer's disease, and certain forms of cancer.^11^,^12^ These diseases constitute the overwhelming majority of health care expenditures in the United States,^13^ and the United Nations Secretary General has declared noncommunicable diseases to be a greater threat to the developing world than acute infectious diseases, including human immunodeficiency virus (HIV).^14^

However, it should be noted that 20% of morbidly obese individuals are metabolically healthy and have normal life spans,^15^,^16^ while up to 40% of adults of normal weight harbor metabolic perturbations typically associated with obesity, including hypertension, dyslipidemia, NAFLD, and CVD.^17^,^18^ Thus, obesity may not be a primary cause of metabolic syndrome; rather, it may be another marker for the underlying metabolic dysfunction that potentially drives its development. Aging does not explain metabolic syndrome either, as young children can manifest these same biochemical processes, especially dyslipidemia, NAFLD, and T2DM.^19^ Thus, metabolic syndrome should be considered a marker for the presence of increased CVD risk rather than a specific phenotype, and metabolic syndrome is now as much a pediatric condition as an adult condition.^20^ How can children experience this degree of metabolic dysfunction?

## Definition of metabolic syndrome

### Adults

The notion that cardiovascular risk factors cluster in certain individuals has been known for several decades. However, it was not until the early 1980s that the relationship between obesity, dyslipidemia (particularly hypertriglyceridemia), and hypertension was recognized.^21^ In the late 1980s–early 1990s, the central roles of insulin resistance (specifically resistance to insulin-stimulated glucose uptake) and abdominal obesity in the syndrome became apparent.^22^ Our current paradigm of metabolic syndrome was established in 1988, when Reaven described the role of insulin resistance in human disease and the interrelation between insulin resistance, hypertension, T2DM, and CVD.^11^ Although Reaven used the term *Syndrome X* to describe the interrelationships of these conditions, many other terms, including the *deadly quartet*, the *cardiometabolic syndrome*, and the *insulin resistance syndrome* have been and continue to be used in the medical literature. Due to the collection of different components, and the reliance of cutoff thresholds of different non-Gaussian distributions, several organizations have established different diagnostic criteria for metabolic syndrome. Nonetheless, the same clustering of CVD risk factors that had been first observed in adults in the early 1920s^23^ became evident in obese children by the mid-1990s.^24^

### Children

Although many attempts have been made to define metabolic syndrome in the pediatric population, to date no consensus definition exists. Indeed, a writing committee of the American Heart Association in 2009 refused to define it (RHL was a committee member).^25^ In 2007, the International Diabetes Federation (IDF) attempted a definition of pediatric metabolic syndrome using age-specific diagnostic criteria^26^ and proposed that metabolic syndrome be considered in (1) children aged 6–10 years who are obese (defined as waist circumference (WC) ≥90th percentile) and have other relevant risk factors (such as family history of cardiometabolic disease) and in (2) children aged 10–16 years who are obese (defined as WC ≥90th percentile) and meet the adult metabolic syndrome criteria for triglycerides (TGs), HDL-cholesterol (HDL-C), blood pressure (BP), and glucose concentrations. Using the IDF definition in the pediatric population and data from the National Health and Nutrition Examination Survey (NHANES) database, the reported prevalence of metabolic syndrome in U.S. adolescents for the period 1999–2004 was approximately 4.5%; it increased with age, was higher among males (6.7%) than females (2.1%), and was highest among Mexican-American adolescents (7.1%).^27^

Several methodological and physiological limitations complicate the establishment of a definition for pediatric metabolic syndrome. For example, children develop transient physiologic insulin resistance during puberty,^28^,^29^ and normal lipid levels vary by age, sex, and race.^30^ Reliance on a fasting blood sample makes diagnosis and detection simple and cheap yet prevents the utilization of a postglucose load sample to detect impaired glucose tolerance (which is a better marker of peripheral insulin resistance in this age group than is a fasting sample). Other barriers include the lack of standardized central obesity measures in children, the lack of normal ranges for insulin assays and concentrations across childhood, and the fact that disturbances in many of the metabolic perturbations associated with metabolic syndrome in children are usually moderate. Children and adolescents with metabolic syndrome may not have the same degree of laboratory abnormalities as those seen in adults.^25^ While laboratory values for some of these phenotypes reflect a risk factor continuum, conventional definitions of metabolic syndrome employ threshold values, creating a false binary system that may obscure important information. One promising approach to overcome this barrier is to use the measurements of metabolic syndrome elements as continuous variables and to sum the *z*-scores of each component in order to quantify the risk.^31^ It has been shown that children of different ethnic backgrounds differ in patterns of lipid partitioning, the major contributor to the development of insulin resistance, and in their metabolic profiles of specific risk markers such as lipids.^32^ Thus, youths of African American origin seem to be “protected” from the syndrome when standard definitions are used, in contrast to the overall worse cardiovascular outcome seen in adults of this ethnic background.

As such, the criteria for metabolic syndrome used in most pediatric studies to date have been variably adapted from adult definitions and standards with the use of available sex- and age-dependent normative values. As in the adult definitions of metabolic syndrome, almost all of them include the following five elements: (1) an elevated TG level, (2) a reduced HDL-C level, (3) a raised BP, (4) an elevated fasting plasma glucose concentration, and (5) an increased WC. Furthermore, most definitions allow for a partial combination of the above factors rather than a requirement that all five be present in order to define metabolic syndrome.

The stability of metabolic syndrome definitions in childhood has been questioned, specifically when assessing the less stringent criteria in obese and normal weight children. Indeed, upon testing normal weight children, the signal to noise ratio of these definitions is low and minor changes induced by normal growth may result in a change in the metabolic syndrome status of an individual.^33^ In contrast, when such definitions are tested in the population at risk (i.e., obese children), weight reduction may still affect their stability yet weight maintenance or gain leads to a stable and reproducible definition.^34^

Currently, 17% of all children and adolescents in the United States are obese,^35^ and childhood obesity is associated with insulin resistance,^36^–^38^ abnormal glucose metabolism,^39^ elevated BP,^40^ dyslipidemia,^41^ inflammation,^27^ and compromised vascular function^42^—all components of metabolic syndrome.^25^ Obesity and its metabolic complications also track from childhood to adulthood.^43^ So, not only is childhood obesity a strong predictor of subsequent obesity, insulin resistance, and dyslipidemia in adulthood, but weight gain in excess of normal growth during childhood is also a determinant of adult cardiovascular risk.^12^

However, the body mass index (BMI), a calculation (kg/m^2^) based on weight and height that is used to define “overweight” and “obesity,” does not account for all variances in insulin sensitivity and cardiometabolic risk.^44^ Furthermore, there is considerable debate as to the metabolic differences between visceral and subcutaneous fat and the role of these fat depots in cardiometabolic disease. As such, obesity does not automatically indicate the presence of metabolic syndrome. Lipid partitioning (i.e., the distribution of fat among its potential depots) is much more related to the metabolic phenotype of obese children and adolescents than the degree of obesity.^10^ Thus, lipid partitioning is a major determinant of peripheral insulin sensitivity and is strongly associated with other metabolic biomarkers such as systemic inflammation and free fatty acid fluxes. These elements determine the metabolic milieu and phenotype of the individual much more than the degree of obesity per se. Again, obesity is a marker for metabolic dysfunction, not the cause.

An early marker of cardiovascular disease in childhood is intimal–medial thickness (IMT), a surrogate of early atherogenesis. When the various published definitions of metabolic syndrome were tested in overweight and obese adolescents along with IMT measurements, only the most conservative definitions were significantly correlated with the degree of IMT, and the presence of impaired glucose tolerance had a strong positive predictive value for the top quartile of IMT.^45^ These observations suggest that the definition of metabolic syndrome should probably be more conservative (e.g., the extreme 5% vs. 10%) in children than in adults, and that the presence of impaired glucose tolerance—-the early sign of altered glucose metabolism—-should be interpreted as a generalized proatherogenic metabolic state. While some investigators suggest that actual carotid plaques (rather than overall arterial wall thickening) are the true expression of atherosclerosis in this age group, carotid plaques have not been sufficiently studied as outcome variables in children. While standard definitions of metabolic syndrome, along with weight changes, have also been shown to predict the development of prediabetes and type 2 diabetes at the age of 24 years,^46^ the presence of impaired glucose tolerance in adolescents (a component of metabolic syndrome in some definitions) is the best predictor of progression to overt diabetes in adolescence.^47^

## Pathogenesis of metabolic syndrome

### Insulin resistance

The association and clustering of T2DM, hypertension, dyslipidemia, and CVD in adults has led to the hypothesis that the various phenotypes of metabolic syndrome arise from a common antecedent. The World Health Organization (WHO) argues that this antecedent is insulin resistance.^11^,^48^–^50^

*Insulin resistance* is defined as the decreased tissue response to insulin-mediated cellular actions and is the inverse of insulin sensitivity. The term insulin resistance, as generally applied, refers to whole-body reduced glucose uptake in response to physiological insulin levels and its consequent effects on glucose and other insulin-driven metabolic pathways. However, it is now clear that not all tissues in such individuals show equal resistance to insulin. Generalized marked insulin resistance results in global metabolic dysfunction, such as Donohue syndrome or Rabson–Mendenhall syndrome. Thus, the insulin resistance of obesity must of necessity affect different tissues and even different signal transduction pathways within the same tissue differently.

#### Hepatic insulin resistance

The liver plays a major role in substrate metabolism and is the primary target of insulin action. After insulin is released from the β cell following a glucose load, it travels directly to the liver via the portal vein, where it binds to the insulin receptor and elicits two key actions at the level of gene transcription. First, insulin stimulates the phosphorylation of FoxO1, preventing it from entering the nucleus^51^,^52^ and diminishing the expression of genes required for gluconeogenesis, principally phosphoenolpyruvate carboxy-kinase and glucose 6-phosphatase. The net effect is diminished hepatic glucose output. Second, insulin activates the transcription factor sterol regulatory element-binding protein (SREBP)-1c, which, in turn, increases the transcription of genes required for fatty acid and TG biosynthesis, most notably ATP-citrate lyase, acetyl-coenzyme A carboxylase, and fatty acid synthase, which together promote the process of *de novo* lipogenesis (DNL). TGs synthesized by DNL are then packaged with apoliprotein B (apoB) into very low-density lipoproteins (VLDL), which are then exported to the periphery for storage. VLDLs can then be utilized by reciprocal activation of lipoprotein lipase (LPL) on the surfaces of endothelial cells in adipose or muscle tissues.^53^

For reasons that remain unclear, insulin resistant subjects typically have selective or dissociated hepatic insulin resistance; that is, they have impaired insulin-mediated glucose homeostasis (mediated by the FoxO1 pathway) but enhanced insulin-mediated hepatic DNL (mediated by the SREBP-1c pathway).^54^ The increase in free fatty acid (FFA) flux within the liver, either by DNL or FFA delivery via the portal vein, impairs hepatic insulin action,^55^,^56^ which, in turn, leads to increases in hepatic glucose output, the synthesis of proinflammatory cytokines, excess TG, low HDL-cholesterol secretion by the liver, and an elevated number of relatively cholesterol-depleted small dense LDL particles.^57^ This intrahepatic accumulation of FFA and lipids is also detrimental to liver insulin sensitivity, as it leads to the generation of toxic lipid-derived metabolites, such as diacyglycerol (DAG), fatty acyl CoA, and ceramides. These, in turn, trigger activation of protein kinase C-ɛ (PKCɛ,) and serine/threonine phosphorylation of IRS-1, which attenuates hepatic insulin signal transduction.^58^

#### Adipose tissue insulin resistance

The expanded adipose tissue mass that accompanies obesity often leads to increased lipolysis and FFA turnover. Normally, insulin inhibits adipose tissue lipolysis; however, in the insulin resistant state, the process is accelerated, leading to increased FFA release into the circulation. Moreover, visceral adipocytes are more sensitive to catecholamine-stimulated lipolysis than subcutaneous adipocytes, further increasing FFA flux.^59^ Macrophages also infiltrate into adipose tissue and contribute to both adipocyte hypertrophy and cytokine release.^60^,^61^ These circulating cytokines also affect insulin action in other tissues, such as liver and muscle.

#### Muscle insulin resistance

Downstream of an insulin resistant liver, increased plasma FFA levels disrupt the glucose-fatty acid or Randle cycle and insulin-mediated glucose uptake by skeletal muscle,^62^,^63^ facilitating the development of hyperglycemia. The ectopic deposition in skeletal muscle of fat as intramyocellular lipid may also play a direct role in the pathogenesis of insulin resistance and metabolic syndrome via lipid metabolite-induced activation of protein PKCɛ with subsequent impairment of insulin signaling.^58^

The two most important biological effectors associated with insulin resistance in childhood are ethnicity and puberty. Studies show that African American, Hispanic, Pima Indian, and Asian children are less insulin sensitive compared to BMI-matched Caucasian children.^64^ The insulin resistance in minority ethnic groups is manifested as lower insulin-stimulated glucose uptake, concomitant with hyperinsulinemia (evidence of increased insulin secretion from the β cell) and decreased insulin clearance.^65^ During puberty, there is a 25–50% decline in insulin sensitivity with recovery when pubertal development is complete.^66^ However, the compensatory increase in insulin secretion during puberty may be blunted in African American and Hispanic youth, thus increasing their risk for T2DM.^67^

### Lipid partitioning

**Figure 1:**
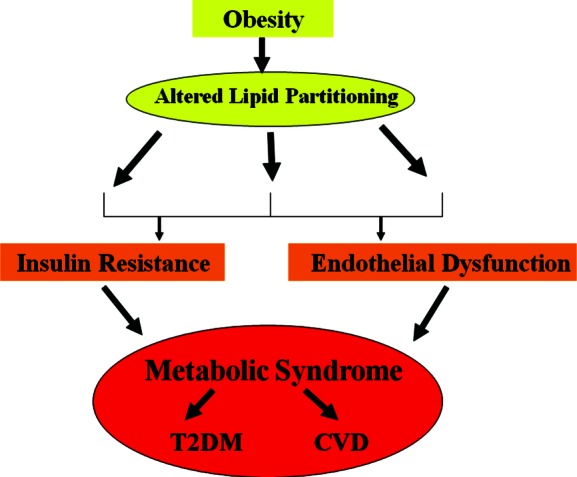
An hypothesis on the relationship between obesity and metabolic syndrome. The metabolic impact of obesity is determined by the pattern of lipid partitioning. Lipid storage in insulin-sensitive tissues, such as liver or muscle, and in the visceral compartment is associated with a typical metabolic profile characterized by elevated free fatty acids and inflammatory cytokines alongside reduced levels of adiponectin. This combination can independently lead to peripheral insulin resistance and to endothelial dysfunction. The combination of insulin resistance and early atherogenesis (manifested as endothelial dysfunction) drives the development of altered glucose metabolism and of cardiovascular disease. (With permission from Ref. 184.)

The term *lipid partitioning* refers to the distribution of body fat in various organs and compartments. The majority of excess fat is stored in its conventional subcutaneous depot, yet other potential storage sites exist as well, such as the intraabdominal (visceral) fat compartment and insulin-responsive tissues such as muscle and liver. Although still debated, one potential etiology of metabolic syndrome is shown in Figure 1. According to this paradigm, the impact of obesity is determined by the pattern of lipid partitioning (i.e., the specific depots in which excess fat is stored). This pattern of lipid storage determines the secretion profile of adipocytokines and its effect on circulating concentrations of inflammatory cytokines and FFA flux. The combined effects of these factors determine the sensitivity of insulin-mediated pathways within target organs (such as muscle and liver) and influence the vascular system by affecting endothelial function.

One hypothesis to explain the relationship between obesity and insulin resistance is the portal-visceral paradigm,^68^ which suggests that increased adiposity causes accumulation of fat in the visceral depot, leading to an increased portal and systemic FFA flux^69^ (Fig. 1). Associations between visceral adiposity, insulin resistance, and comorbidities have been demonstrated across most age groups and ethnicities.^70^ However, studies of *in vivo* FFA fluxes from the visceral and the subcutaneous truncal and abdominal depots have failed to demonstrate a substantial difference in net fluxes between these depots.^71^

Subcutaneous fat, which does not drain into the portal system, is strongly related to insulin resistance in both healthy obese and diabetic men.^72^ Similarly, truncal subcutaneous fat mass has been demonstrated to independently predict insulin resistance in obese women. Visceral and subcutaneous fat differ in their biologic properties,^73^ as visceral fat is more resistant to insulin and has increased sensitivity to catecholamines. These observations emphasize that both visceral and subcutaneous abdominal fat can contribute to insulin resistance, possibly by different mechanisms.^74^

Recent studies performed in obese adolescents highlight the fact that the ratio of visceral to subcutaneous fat, rather than the absolute quantity of body fat, may be the determinant of metabolic impact. Indeed, obese adolescents with a high ratio, relative to even more obese individuals with lower ratios, demonstrate a markedly adverse metabolic phenotype of severe insulin resistance and alterations in glucose and lipid metabolism.^75^ Moreover, intrahepatic fat, while strongly associated with high levels of visceral fat, is independently associated with the insulin-resistant state in obese adolescents, independent of all other fat depots.^76^

An alternative theory to explain the relationship between obesity and insulin resistance is the “ectopic lipid deposition” paradigm.^77^ This theory is based on the observations that lipid content of insulin responsive tissues such as liver and/or muscle is increased in obesity and in T2DM and is a strong predictor of insulin resistance.^78^,^79^ Moreover, in conditions such as lipodystrophies, all fat is stored in liver and muscle due to lack of subcutaneous fat tissue, causing severe insulin resistance and diabetes.^80^ In obese adults (BMI > 30 kg/m^2^), muscle attenuation on computed tomography (CT), representing lipid content, is a stronger predictor of insulin resistance than is visceral fat.^81^ Studies performed *in vivo* using ^1^H-NMR spectroscopy also demonstrated increased intramyocellular lipid (IMCL) content to be a strong determinant of insulin resistance in adults^82^ and in obese adolescents.^83^ Furthermore, lipid deposition in hepatocytes and the production of intrahepatocellular lipid (IHCL) are highly predictive of insulin resistance, even more so than visceral fat.^84^ Thus, morbidity may begin when the subcutaneous fat depot reaches its storage capacity and begins to shunt lipid to ectopic tissues, such as liver and muscle, leading to peripheral insulin resistance,^85^ or alternatively when the liver or muscle accumulates fat produced by DNL in response to dietary factors (see below).

Another postulated cause of IMCL and IHCL accumulation is not lipid shunting from adipose depots, but rather *de novo* lipogenesis along with a reduction of β-oxidation of fat^86^ due to low aerobic capacity, a reduced number or malfunction of mitochondria, or reduced sympathetic tone. The effects of IMCL or IHCL accumulation on peripheral insulin sensitivity are postulated to result from an alteration of the insulin signal transduction pathway, caused by derivates of fat such as long chain fatty acyl-CoA and DAG in the hepatocyte or myocyte. In muscle, these derivatives activate the serine/threonine kinase cascade and cause serine phosphorylation of IRS-1, which inhibits insulin signaling.^87^ A comparable mechanism has been demonstrated in the liver, where accumulation of lipids, DAG in particular, activates the inflammatory cascade by inducing c-jun N-terminal kinase (JNK-1), which causes serine rather than tyrosine phosphorylation of IRS-1 and leads to inhibition of hepatic insulin signaling.^88^,^89^

### Adipocytokines

#### Leptin

The discovery of leptin in 1994 dramatically changed the view of adipose tissue in the regulation of energy balance.^90^ Adipocytes secrete several proteins that act as regulators of glucose and lipid homeostasis.^91^ These proteins have been collectively referred to as adipocytokines because of their structural similarities with cytokines. Circulating leptin levels serve as an adiposity sensor to protect against starvation and correlate with the degree of obesity. Leptin probably has a permissive role in high-energy metabolic processes such as puberty, ovulation, and pregnancy, but its role in states of energy excess is less known. In obesity, the development of leptin resistance may result in a breakdown of the normal partitioning of surplus lipids in the adipocyte compartment.^92^

#### Adiponectin

Adiponectin is peculiar because, in contrast to the other adipocytokines, its level is reduced in obesity.^93^ The adiponectin gene is located on chromosome 3q27, a location previously linked to the development of T2DM and metabolic syndrome. Several single nucleotide polymorphisms (SNPs) in the adiponectin gene have been reported to be associated with the development of T2DM in populations around the world, suggesting that adiponectin plays a major role in glucose and lipid metabolism.^94^ Adiponectin circulates in plasma in three major forms: a low molecular weight trimer, a middle molecular weight hexamer, and a high molecular weight 12- to 18-mer.^95^ Circulating plasma adiponectin concentrations demonstrate a sexual dimorphism (females have higher concentrations), suggesting a role for sex hormones in the regulation of adiponectin production or clearance. Dietary factors such as linoleic acid or fish oil versus a high carbohydrate diet or increased oxidative stress have been shown to increase or decrease adiponectin concentrations, respectively. These observations suggest that the circulating levels of adiponectin are regulated by complex interactions between genetic and environmental factors.^96^

Two adiponectin receptors, named ADIPOR1 and ADIPOR2, have been characterized. ADIPOR1 is expressed in numerous tissues including muscle, while ADIPOR2 is mostly restricted to the liver. Both receptors are bound to the cell membrane, yet are unique in comparison to other G protein–coupled receptors in the fact that the C-terminus is extracellular while the N-terminus is intracellular.^97^ Both ADIPOR1 and ADIPOR2 are receptors for the globular head of adiponectin and serve as initiators of signal transduction pathways that lead to increased peroxisome proliferator-activated receptor (PPAR)-α and AMP kinase activities, which promote glucose uptake and fatty acid oxidation. Adiponectin has also been shown to have potent antiatherogenic functions, as it accumulates in the subendothelial space of injured vascular walls, reducing the expression of adhesion molecules and the recruitment of macrophages.^98^

Studies in obese children and adolescents have shown that adiponectin levels are inversely related to the degree of obesity, insulin resistance, visceral adiposity, IHCL, and IMCL, while weight loss increases adiponectin concentrations. A fall in adiponectin levels has also been shown to coincide with the onset of insulin resistance^99^ and the development of diabetes in monkeys.^100^ All of these observations, along with human clinical data, support a pivotal role for adiponectin in the prevention of the comorbidities of metabolic syndrome.

#### Inflammatory cytokines

Accumulating evidence indicates that obesity is associated with subclinical chronic inflammation.^101^ Thus, the adipose tissue serves not merely as a simple reservoir of energy, but also as an active secretory organ releasing many peptides, including inflammatory cytokines, into the circulation. In obesity, the balance between these peptides is altered, such that larger adipocytes and macrophages embedded within them produce more inflammatory cytokines (i.e., TNF-α and IL-6) and fewer anti-inflammatory peptides such as adiponectin.^102^ One theory posits that as energy accumulates in adipocytes, the perilipin border of the fat vacuole breaks down, causing the adipocyte to die.^103^ Cell death then recruits macrophages in the adipose tissue, especially the visceral compartment, that in the process of clearing debris also secrete inflammatory cytokines, initiating a proinflammatory cascade that predates and possibly drives the development of systemic insulin resistance, diabetes, and endothelial dysfunction.^104^,^105^ Systemic concentrations of C-reactive protein (CRP) and IL-6, two major markers of inflammation, are increased in obese children and adolescents. Indeed, CRP levels within the high-normal range have been shown to predict CVD^106^ and the development of T2DM^107^ in adults. Elevated levels of CRP also correlate with other components of metabolic syndrome in obese children.^108^,^109^ Thus, inflammation may be one of the links between obesity and insulin resistance, and may also promote endothelial dysfunction and early atherogenesis.

Most of the aforementioned molecules have been associated with elements of metabolic syndrome and its characteristic pattern of lipid partitioning. Specifically, low adiponectin levels have been associated with insulin resistance, low-grade inflammation and increased intramyocellular fat.^110^ High leptin concentrations have also been shown to be associated with metabolic syndrome in adults.^111^ Moreover, factor analyses of plasma leptin concentrations and the variables that are considered relevant to metabolic syndrome revealed a clustering of plasma leptin concentrations with insulin resistance and hyperinsulinemia.^112^

### Reactive oxygen species

**Figure 2:**
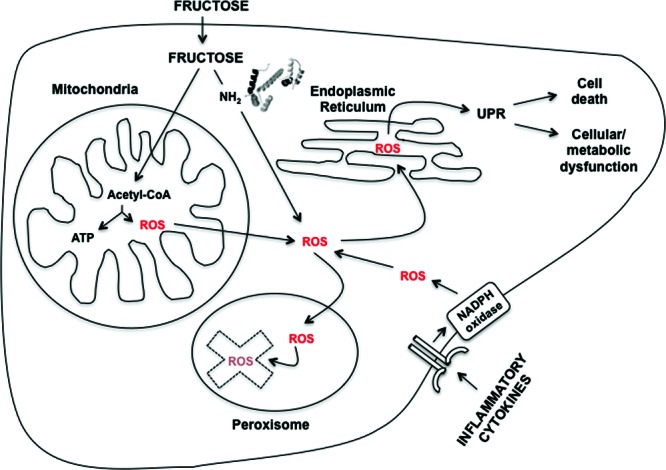
Mechanisms of subcellular metabolic dysfunction, using fructose as an example. The formation of acetyl-CoA leads to lipid deposition and activation of inflammatory pathways, including serine phosphorylation of IRS-1, which leads to insulin resistance. Furthermore, metabolic processing in the mitochondria, the glycation of protein ɛ-amino groups via the Maillard reaction, and circulating inflammatory cytokines due to their receptor-mediated activation of NADPH oxidase, all increase intracellular levels of ROS. In the absence of sufficient peroxisomal quenching and degradation, the ROS moieties lead to endoplasmic reticulum stress, promoting the unfolded protein response, and cause either cell death (apoptosis) or cellular/metabolic dysfunction. (With permission from Ref. 18.) Courtesy of the American Academy of Pediatrics. Abbreviations: ATP, adenosine triphosphate; CoA, coenzyme A; JNK-1, c-jun N-terminal kinase 1; NADPH, nicotinamide adenine dinucleotide phosphate; PKCɛ, protein kinase C-ɛ; pSer-IRS-1, serine phosphorylated IRS-1; ROS, reactive oxygen species; UPR, unfolded protein response.

The free radical theory holds that an imbalance between reactive oxygen species (ROS) generation and antioxidant defenses is a major factor in the determination of lipid peroxidation and protein misfolding, with resultant DNA and cellular damage.^113^ Excessive intracellular ROS formation occurs via three pathways: (1) inflammatory cytokines derived from visceral fat accumulation;^114^ (2) dysfunctional mitochondrial energetics;^115^ and (3) glycation (see below). Excessive nutrient processing by mitochondria can result in uncoupling of oxidative phosphorylation and an increased generation of ROS; this, in turn, leads to altered mitochondrial function and further ROS generation.^116^ ROS accumulation can also impair endoplasmic reticulum (ER) function, causing ER stress and the compensatory unfolded protein response (UPR). The UPR can itself be overwhelmed by persistent excessive nutrient processing and ROS generation, leading to cellular shutdown, defective insulin secretion, and T2DM^117^,^118^ (Fig. 2).

As ROS generation is an inherent by-product of cellular metabolism, endogenous cellular antioxidants (e.g., catalase and glutathione) quench the ROS before they have a chance to promote peroxidation. These antioxidants are found primarily in peroxisomes, which abut the mitochondria, and support ROS processing. A reduction in peroxisomal activity results in mitochondrial dysfunction and ER stress. Cytokines such as TNF-α can reduce peroxisomal number and function, rendering cells even more vulnerable.^119^,^120^

### ER stress and the UPR

Excessive ROS that are not quenched by peroxisomes find their way to the adjacent ER, where they alter the redox environment crucial for proper protein folding.^121^ Accumulation of ROS and misfolded proteins within the ER activates the UPR,^122^ which is designed to decrease protein synthesis in order to allow for their clearance.^123^ However, excessive ROS levels impair the ability of a cell to clear misfolded proteins and activate the enzyme caspase-3, leading to even further ROS generation, apoptosis, and cellular demise.^124^ ER stress in the liver is a specific mechanism of hepatic injury in NAFLD,^125^ and ER stress in the pancreas reduces β-cell number and promotes diabetes.^126^

## Environmental antecedents

If obesity is not the cause of metabolic syndrome, what is? Although many investigators have searched for genetic predispositions, it has been estimated that only 10% of metabolic syndrome cases can be explained by genetics.^127^ Alternatively, numerous environmental correlates have been identified. However, most of these associations are cross-sectional rather than longitudinal; and in many cases mechanisms remain undiscovered.^128^

### Stress and cortisol

In humans, elevated cortisol or markers of hypothalamic–pituitary–adrenal (HPA) axis dysregulation correlate with abdominal fat distribution and metabolic syndrome.^129^ Although circulating cortisol is clearly important in determining visceral adiposity, the reduction of circulating cortisone to cortisol within visceral fat tissue by the enzyme 11β-hydroxysteroid dehydrogenase-1 (11β-HSD1) has also recently been linked to metabolic syndrome.^130^,^131^ These data suggest that cortisol is important both in increasing visceral adiposity and in promoting metabolic syndrome.

In adults, job stress and depression stress are associated with increased cortisol secretion,^132^ which leads to insulin resistance and metabolic syndrome. Psychosocial stresses correlate with an elevated risk of myocardial infarction in adults,^133^ and it is assumed that such patients exhibit increased HPA axis activation.^134^ Even exogenous glucocorticoid administration is a risk factor for CVD events.^135^ Evidence of associations between elevated cortisol and psychological distress with abdominal fat distribution in adults is compelling. For instance, urinary glucocorticoid excretion is linked to aspects of metabolic syndrome, including BP, fasting glucose, insulin, and WC.^129^ The role of cortisol in mediating visceral fat accumulation, insulin resistance, and T2DM has been elegantly demonstrated by transgenic knockout and overexpression models of 11β-HSD1.^130^,^131^ It appears that some individuals are high responders to stress stimuli and demonstrate higher cortisol secretion. These individuals seem more prone to alterations in satiety recognition and consume larger amounts of calories following the stress exposure. Thus, cortisol appears to be important both in increasing visceral adiposity and promoting metabolic syndrome—equivalent to Cushing's syndrome of the abdomen.^136^ However, the role of stress and cortisol in childhood obesity is currently speculative.

### Sleep deprivation

Americans get significantly less sleep than they did three decades ago. Adults in the United States currently average less than seven hours of sleep per night—almost two hours less than in 1980—and about one-third of them get less than six hours per night.^137^ Analyses of data from NHANES I revealed that adults (32–49 years old) who slept less than seven hours per night were more likely to be obese five to eight years later than those who slept seven or more hours.^138^ Similarly, a 13-year prospective cohort study in which participants were interviewed at 27, 29, 34, and 40 years of age found that sleep duration correlated negatively with obesity.^139^ The link between short sleep duration and obesity has also been observed among children.^140^ Like adults, increasing numbers of children are chronically sleep deprived. This is especially true of obese children, who have been found to get less sleep than normal weight children. In addition to its other effects, sleep is one of the most powerful cross-sectional^141^ and longitudinal^142^ predictors of obesity in prepubertal children. Obesity is a major risk factor for OSA at all ages and OSA is tightly linked mechanistically to insulin resistance and low-grade inflammation, the drivers of metabolic syndrome.^143^ Although relatively little is known about the mechanism(s) for the sleep–obesity relationship,^144^ especially among children, there are reasons to suspect increased stress and altered activity of various hormones, such as leptin, ghrelin, and cortisol.

### Dietary factors

While the primary focus regarding obesity has been on total calories ingested, an emerging evidence base suggests that the quality of those calories play an important role in the pathogenesis of the metabolic syndrome by increasing hepatic insulin resistance and/or increasing ROS formation.

#### Dietary fat versus carbohydrate

Fat is generally considered more obesogenic than other macronutrients, given that it has greater energy density, is highly palatable, and is more effectively converted to body fat.^145^ A high-fat diet induces decreased thermogenesis and a higher positive fat balance than an isocaloric and isoproteic low-fat meal.^146^ Excessive fat intake is believed to cause weight gain,^147^ but the relationship between dietary fat intake and childhood adiposity remains controversial.^148^

The prevalence of overweight individuals in the United States has increased despite a decreased percentage of dietary energy derived from fat. A meta-analysis of 12 studies in overweight or obese adults who were given dietary advice on a low-fat diet and followed for 6–18 months suggested that low-fat diets are no more effective than calorie-restricted diets for long-term weight loss.^149^ Similarly, in children, total fat consumption expressed as a percentage of energy intake has decreased.^150^ This decrease in fat consumption has been paralleled by an increase in total energy intake, mostly in the form of carbohydrates. Much of this imbalance is attributed to changing beverage consumption patterns, characterized by declining milk intakes and substantial increases in soft-drink consumption,^151^ which may have its own etiopathogenesis to metabolic syndrome (see below). Moreover, most interventions with a low-fat, heart-healthy diet have not been successful in childhood overweight prevention.^152^

Reduction in carbohydrate intake is taken to the extreme in the Atkins diet, which restricts adult subjects to less than 25 gm/day of ingested carbohydrate. Adult evaluations of the diet have been disappointing long term, 153,154 and there is currently a single study regarding the effect of the modified carbohydrate diet on obesity in children or adolescents that demonstrated short-term efficacy yet difficulties in adherence.^155^ However, it should be noted that the ketogenic diet used for seizure control is similar in composition to the Atkins diet. A two-year study of the ketogenic diet demonstrated persistent decreases in weight *z*-scores in children who were above average upon diet initiation, without significant compromise in general nutrition or in height.^156^

#### Fructose

The most commonly used sweetener in the U.S. diet is the disaccharide sucrose (e.g., table sugar), which contains 50% fructose and 50% glucose. However, in North America and many other countries, nondiet soft drinks are sweetened with high-fructose corn syrup (HFCS), which contains up to 55% of the monosaccharide fructose. HFCS is found in processed foods ranging from soft drinks and candy bars to crackers, hot dog buns, and ketchup. Average daily fructose consumption has increased by over 25% over the past 30 years, and the growing dependence on fructose in the Western diet may be fueling the obesity and T2DM epidemics.^157^ The highest fructose loads are soda (1.7 gm/oz) and juice (1.8 gm/oz). Although soda has received most of the attention,^158^,^159^ high fruit juice intake is also associated with childhood obesity, especially in lower income families.^160^

Animal models demonstrate that high-fructose diets lead to increased energy intake, decreased resting energy expenditure, excess fat deposition, and insulin resistance,^161^,^162^ which also suggest that fructose consumption is playing a role in the epidemics of insulin resistance, obesity, and T2DM in humans.^163^,^164^

**Figure 3:**
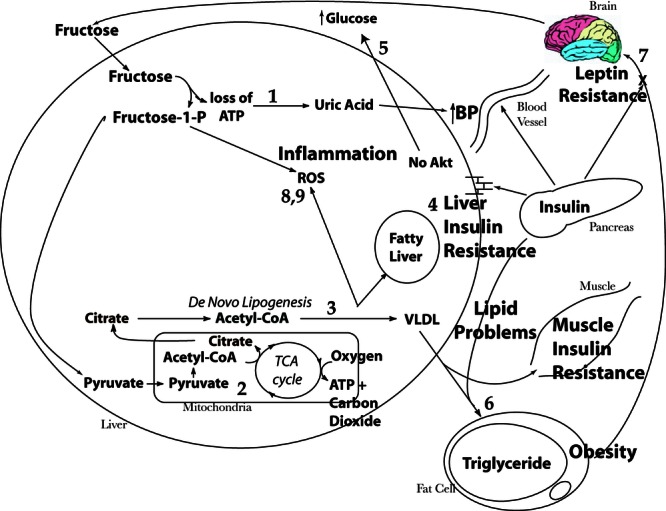
Hepatic fructose metabolism. In contrast to glucose, fructose induces (1) substrate-dependent hepatocellular phosphate depletion, which increases uric acid and contributes to hypertension through inhibition of endothelial nitric oxide synthase and reduction of nitric oxide (NO); (2) excess citrate production; (3) stimulation of *de novo* lipogenesis and excess production of VLDL and serum TG, promoting dyslipidemia; (4) accumulation of intrahepatic lipid droplets, promoting hepatic steatosis; (5) lack of phosphorylation of FoxO1, leading to increased gluconeogenesis; (6) delivery of triglycerides to muscle, promoting muscle insulin resistance; (7) CNS hyperinsulinemia, which antagonizes leptin signaling and promotes continued energy intake; (8) JNK-1 activation, which causes serine phosphorylation of the hepatic insulin receptor rendering it inactive and contributing to hepatic insulin resistance; and (9) production of reactive oxygen species (ROS), which lead to protein instability.

Fructose in the gut is transported into the enterocyte via the fructose transporter, Glut5, independent of ATP hydrolysis and sodium absorption. Once inside the enterocyte, a small portion of the fructose load is converted to lactic acid and released in the portal circulation, another small portion may also be converted to glucose. However, the majority of ingested fructose is secreted into the portal circulation and delivered to the liver. There, fructose is rapidly metabolized to fructose-1-phosphate (F1P) via fructokinase, an insulin-independent process that also bypasses the negative feedback regulation of phosphofructokinase in the glycolytic pathway. Thus, fructose metabolism generates lipogenic substrates (e.g., glyceraldehyde-3-phosphate and acetyl-CoA) in an unregulated fashion, which are delivered straight to the mitochondria. This excessive mitochondrial substrate then drives hepatic DNL, which can then overwhelm apoB and the lipid export machinery, leading to intrahepatic lipid deposition and steatosis.^165^ Hepatic DNL also limits further fatty acid oxidation in the liver via excess production of malonyl-CoA, which reduces entry of fatty acids into the mitochondria by inhibiting carnitine palmitoyl transferase-1 (CPT-1). F1P activates dual-specificity mitogen-activated protein kinase 7 (MKK7), which subsequently stimulates JNK-1, a hepatic enzyme considered to act as a bridge between hepatic metabolism and inflammation.^166^ In addition, the lipogenic intermediate DAG (formed during fructose metabolism in the liver) activates PKC-ɛ, which leads to serine phosphorylation of IRS-1 that inactivates it and leads to hepatic insulin resistance.^165^ This impairs insulin-mediated phosphorylation of FoxO1, leading to increased expression of the genes required for gluconeogenesis and promoting increased hepatic glucose output, which subsequently contribute to hyperglycemia and the development of T2DM. The excess TGs secreted from the liver into the circulation as fat-laden VLDL particles following the ingestion of fructose, coupled with a fructose-induced reduction in LPL activity, cause sustained postprandial dyslipidemia, thereby augmenting the risk for CVD^167^,^168^ (Fig. 3).

Fructose also does not suppress secretion of the so-called hunger hormone ghrelin, levels of which correlate with perceived hunger.^169^ In sum, fructose consumption has metabolic and hormonal consequences that facilitate development of obesity and the metabolic syndrome.^164^

Due to its unique stereochemistry, the ring form of fructose is under a great deal of ionic strain, which favors the linear form of the molecule, exposing the reactive 2-keto group, which can readily engage in the nonenzymatic fructosylation of exposed amino moieties of proteins via the Maillard reaction in the same way that the 1-aldehyde position of glucose is reactive.^165^ Each Maillard reaction generates one ROS, which must be quenched by an antioxidant at the risk of cellular damage. In an *in vitro* study, incubation of hepatocytes with fructose yielded no direct damage; however, when these hepatocytes were preincubated with sublethal doses of hydrogen peroxide to reduce their peroxisomal ROS-quenching ability, fructose then became as hepatotoxic as other organic aldehydes.^170^ Furthermore, an *in vivo* study in antioxidant-deficient mice demonstrated that intrahepatic lipid toxicity and hepatocellular death occurred following sucrose administration.^171^ These data thus suggest that excessive ROS, in combination with micronutrient insufficiencies that impair antioxidant reserves, can lead to cellular damage and promote the metabolic syndrome.

#### Branched-chain amino acids

Branched-chain amino acids (BCAAs: valine, leucine, and isoleucine) are essential amino acids that account for more than 20% of the amino acids in the typical Western diet.^172^ Although normally utilized for protein biosynthesis and cell growth, when provided in excess they are diverted away from protein synthesis and toward energy utilization.^173^

In the liver, BCAAs increase transcription of ChREBP and SREBP-1c,^174^ facilitating DNL. Furthermore, BCAAs limit insulin-induced PI3K signaling and stimulate the activation of the mammalian target of rapamycin (mTOR), promoting the serine phosphorylation of IRS-1 and impairment of insulin signaling. In addition, just as there are obesity-related changes in adipokines and cardiovascular risk markers, there also appear to be obesity-associated changes in BCAA metabolism and resulting serum levels. In particular, valine and leucine/isoleucine levels have been reported to be 20% and 14% higher, respectively, in obese compared to lean subjects.^173^ Mechanistically, this appears to be accounted for by a high rate of flux through the BCAA catabolic pathway, resulting in the increased production of alanine. Since alanine is a highly gluconeogenic amino acid, increased BCAA catabolism may thus contribute to increased hepatic glucose output.^175^ Furthermore, the increased α-ketoacids generated by increased flux of the BCAAs through their catabolic pathways also potentially suppress mitochondrial β-oxidation.

Furthermore, chronic BCAA elevation impairs the transport of aromatic amino acids into the brain; the reduced production of serotonin (derived from tryptophan) and catecholamines (derived from phenylalanine and tyrosine) may drive hunger.^173^ The BCAA overload hypothesis suggests that in the context of a dietary pattern that includes high fat consumption, BCAAs may make an independent contribution to the development of insulin resistance, a hypothesis supported by metabolomic studies demonstrating high BCAA levels in normoglycemic individuals that subsequently develop insulin resistance and diabetes.^176^,^177^ Although the data supporting a role of BCAAs in the development of metabolic syndrome components are currently only correlative in nature, the BCAA overload hypothesis is intriguing and relevant.

#### Ethanol

Although adult epidemiological studies associate light to moderate ethanol consumption with improved insulin sensitivity and wine consumption with reduced cardiovascular risk, other cross-sectional and prospective studies implicate a dose-dependent effect of alcohol in metabolic syndrome, and suggest that chronic consumption of large amounts of ethanol worsen insulin sensitivity. Ethanol bypasses glycolysis by being converted by alcohol dehydrogenase-1B into acetaldehyde, which promotes ROS formation and must also be quenched by hepatic antioxidants such as glutathione or ascorbic acid to prevent cellular damage. Acetaldehyde is then metabolized by the enzyme aldehyde dehydrogenase-2 to acetic acid, which, in turn, is metabolized by the enzyme acyl-CoA synthetase short-chain family member 2 to form acetyl-CoA. The acetyl-CoA can then enter the mitochondria; or, in the presence of other caloric substrates, it is preferentially used for the synthesis of fatty acids through DNL. The excess malonyl-CoA produced from ethanol metabolism inhibits CPT-1, limiting mitochondrial fatty acid β-oxidation. Ethanol also blocks fatty acid β-oxidation by inhibiting PPAR-α, which suppresses microsomal triglyceride transfer protein, thereby altering the liver's lipid export machinery.^178^–^181^ Buildup of intrahepatic lipid metabolites leads to subsequent activation of the enzyme JNK-1 and serine phosphorylation of IRS-1, driving further hepatic insulin resistance. Thus, ethanol metabolism results in intrahepatic lipid accumulation and liver injury, driving hepatic insulin resistance and promoting metabolic syndrome.^182^ However, while clearly a concern in adults, the likelihood that ethanol contributes significantly to metabolic syndrome in children is highly suspect.

How are these four dietary foodstuffs similar? They share the following biochemical properties: (1) they are metabolized for energy primarily within the liver; (2) they are not insulin regulated; and (3) they do not have a threshold mechanism to form glycogen for storage. Although the kinetics of their metabolism may differ, virtually all their intermediates are delivered directly to the mitochondria, which cannot process the volume of substrate, resulting in a backlog of metabolic intermediates, ROS generation, excessive DNL, and impaired β-oxidation, driving insulin resistance and the downstream comorbidities of the metabolic syndrome.

## Conclusions

Metabolic syndrome is a complex phenotype that correlates with obesity, but nonetheless appears to be distinct from it. The fact that children can develop metabolic syndrome suggests that while obesity and aging contribute to the syndrome, it is unlikely that they are initiating factors. The fact that children around the world are getting heavier^183^ and developing the syndrome argues against genetics. More likely, the environment plays a major role, in particular the typical Western diet, which has now been adopted globally due to palatability and price. This review outlines the potential instigators and their pathophysiological mechanisms, which starts with mitochondrial overload and results in *de novo* lipogenesis, insulin resistance, ROS formation, peroxisomal dysfunction, and ER stress and the UPR.^184^,^185^ Sadly, there does not appear to be a likely drug target in this pathway to reduce mitochondrial overload. While medications can treat the various disease states associated with metabolic syndrome, prevention is paramount. We proffer the notion that an overhaul of the typical Western diet will be required to beat metabolic syndrome once and for all.
